# Exploring the role of voluntary disease schemes on UK farmer bio-security behaviours: Findings from the Norfolk-Suffolk Bovine Viral Diarrhoea control scheme

**DOI:** 10.1371/journal.pone.0179877

**Published:** 2018-02-12

**Authors:** Lena Azbel-Jackson, Claire Heffernan, George Gunn, Joe Brownlie

**Affiliations:** 1 Livestock Development Group, School of Agriculture, Policy and Development, University of Reading, Reading, United Kingdom; 2 London International Development Centre, London, United Kingdom; 3 Future Farming Systems, Epidemiology Research Unit, Scotlands’ Rural College, Drummondhill, Inverness, United Kingdom; 4 The Royal Veterinary College, Hawkshead Lane, Hatfield, Hertfordshire, United Kingdom; TNO, NETHERLANDS

## Abstract

The article describes the influence of a disease control scheme (the Norfolk-Suffolk Bovine Viral Diarrhoea Disease (BVD) Eradication scheme) on farmers' bio-security attitudes and behaviours. In 2010, a survey of 100 cattle farmers (53 scheme members vs. 47 out of scheme farmers) was undertaken among cattle farmers residing in Norfolk and Suffolk counties in the UK. A cross-sectional independent measures design was employed. The main analytical tool was content analysis. The following variables at the farmer-level were explored: the specific BVD control measures adopted, livestock disease priorities, motivation for scheme membership, wider knowledge acquisition, biosecurity behaviours employed and training course attendance. The findings suggest that participation in the BVD scheme improved farmers' perception of the scheme benefits and participation in training courses. However, no association was found between the taking part in the BVD scheme and livestock disease priorities or motivation for scheme participation, or knowledge about BVD bio-security measures employed. Equally importantly, scheme membership did appear to influence the importance accorded specific bio-security measures. Yet such ranking did not appear to reflect the actual behaviours undertaken. As such, disease control efforts alone while necessary, are insufficient. Rather, to enhance farmer bio-security behaviours significant effort must be made to address underlying attitudes to the specific disease threat involved.

## Introduction

Bovine Viral Diarrhoea (BVD) is a viral disease of cattle with large impacts on herd productivity and reproduction [1]. It is estimated that more than 90% of UK herds have been exposed to the virus [2]. Numerous studies show that BVD has considerable economic consequences at the farm level [1,3–5]. Scottish Government estimates suggest that the eradication of BVD would be worth from £50 to £80 million in increased outputs and reduced costs for the livestock industry over the next 10 years [6]. Despite the high cost of the disease, efforts at BVD control and eradication, at the farm level, are considered patchy at best [7]. In response, the Scottish Government has recently legislated a mandatory control scheme [6]. While no such legislation is planned for England, in 2016 the industry-led BVD Free England scheme was initiated (www.bvdfree.org.uk).

Previous research has shown that there are a number of factors that influence farm-level biosecurity behaviours. Key factors include farmer age [8–10]) education levels [11] perception of the risk/impact of a particular disease [12–14] and the overall cost of implementing biosecurity measures [15–18]. Attitudes toward specific bio-security measures themselves have also been found to be important [13, 14,19]. Access to available sources of information on biosecurity measures and animal health issues ([13, 14, 20] and attitudes towards these sources [13, 14, 21, 22] also play a role. Finally, discussions of bio-security measures with advisors or veterinary surgeons [13, 14, 23] can drive specific behaviours.

The impact of the health schemes on the implementation of bio-security behaviours by farmers is less clear. For instance, Heffernan and colleagues reported that participation in the schemes had no effect on farmer’s ability to work together, or knowledge uptake, or attitude toward biosecurity regulation [13]. However, in an article which examined the impact of a priori determinants of bio-security behaviour among UK farmers Toma reported ([14]: pg. 332):

… another strong influence on [bio-security] behaviour comes from membership in a cattle/sheep health scheme, which explained 5% and 25% of the variance in behaviour…This might suggest that farmers who are members in cattle and/or sheep schemes are likely to apply more biosecurity measures on their farms.

In an environment of increasing resource scarcity, there is an urgent need to find cost effective solutions to animal health threats. Farmer-led disease control schemes are one such option. Therefore, in the following article, the authors examine differences in bio-security attitudes and behaviours among two groups of farmers residing in Norfolk and Suffolk: those enrolled in a BVD scheme (The Norfolk and Suffolk BVD Eradication Scheme) vs. those who are not. We investigated a range of factors hypothesized to have an influence on bio-security behaviours: BVD control measures employed, livestock disease priorities, motivation for scheme membership, perception of the scheme benefits and wider knowledge acquisition.

The Norfolk-Suffolk Bovine Viral Diarrhoea Disease (BVD) Eradication scheme was initiated in 2007. The aim was to declare Norfolk and Suffolk counties BVD free. According to the scheme promotional material this aim could be achieved by supporting farmers in detecting BVD in their livestock and by eliminating the persistently infected (PI) animals [24]. In this manner, the explicit goal of the scheme did not relate to the formation of farmers groups or group participation per se. Rather, local veterinarians led the scheme and encouraged farmers to participate. Further, participation in the scheme was voluntary. Farmers, who were part of the scheme, were expected to test their cattle annually for BVD. Data as to the overall success or failure of the scheme was not available to the principle authors at the time of data collection.

## Materials and methods

### Design and participants

The authors employed a cross-sectional design, in total, 100 farmers (*N* In-Scheme = 53, *N* Out of Scheme = 47) were interviewed across the Norfolk and Suffolk counties in UK.

Data collection activities were undertaken over a two-week period in December 2010. Convenience sampling was utilized to target members of the Norfolk/Suffolk BVD Eradication Scheme. While 12 veterinary practices across the two counties implemented the scheme, when contacted local veterinarians from only three of the practices identified participants for inclusion in the study. Of the ten study participants identified in this manner, five agreed to be interviewed. Latterly, a list of scheme farmers was supplied by the Royal Veterinary College (an implementer of the Norfolk/Suffolk scheme). All scheme members were contacted to participate (n = 101), 48 subsequently agreed. Scheme farmers were then interviewed in person (n = 38) and via the telephone (n = 10).

Non-scheme farmers were interviewed at the Norwich Livestock Market, Norwich and Newark Livestock Market in Newark, Nottinghamshire. These markets were selected solely due to their geographic proximity to the schemes. Ultimately participation in the study by farmers in both groups relied on the good will of the individuals involved. However, farmers who did not reside in the geographic area of the schemes, were excluded from participating.

To lower observer bias, the same team interviewed in-scheme vs. out of scheme farmers. All team members underwent a half-day training in qualitative data collection techniques (n = 4). Key features included the elements of a quality interview from the ‘word for word’ capturing of farmers’ narratives to the differences in probing vs. prompting responses, to tools for lowering observer bias and establishing a successful rapport with participants. Mock interviews using the questionnaire were then filmed to ensure that all researchers understood the aim of the questions and to identify any areas of an individual’s interview technique that could be improved.

Questionnaires were semi-structured and farmers were asked to describe different elements of bio-security in their own words (enumerators were responsible for transcribing the narratives, as interviews were not recorded). Participants were shown the questionnaires prior to the exchange and were provided with a participant consent form detailing the aims of the research and confirming that all responses would be anonymised. For phone interviews, the component parts of the questionnaire were described ex-ante to farmers. Potential interviewees were then asked to provide verbal consent for their participation in the project. Those agreeing were then asked to sign the above statement. The specific procedures for data collection and participant consent were in full compliance with the ethical guidelines and standards for human participant research as detailed and further approved by the Research Ethics Committee, School of Agriculture, Policy and Development, University of Reading.

### Data analysis

A content analysis was undertaken on the narratives produced by the interviewees. Content analysis is a technique for systematically describing data [20]. It provides quantitative and qualitative descriptions of the information collected. In content analysis, the interview narratives were broken down into themes, in this case the five variables comprising the analysis: bio-security measures employed with a focus on BVD control measures, livestock disease priorities, motivation for scheme membership, perception of scheme benefits and knowledge acquisition. To explore the themes, farmers were asked a range of both open-ended and closed questions. To lower researcher bias, coding was non-inductive i.e. rather applying pre-existing codes, which may conform to researcher expectations or confirm existing beliefs, in this study the topics and subsequent codes for qualitative questions emerged from the data itself.

Statistical analysis was performed using IMB SPSS Statistics 22 software (IBM, 2014).The results were then summarized where the independent variable was the farmer group (in scheme farmers vs. out of scheme farmers) and the dependent variable was the biosecurity behaviour indicators (BVD control measures employed, livestock disease priorities, motivation for scheme membership, wider knowledge acquisition, the priority of biosecurity behaviours employed and the frequency of training courses attended). Because of the nonparametric nature of the biosecurity behaviours indicators data, chi-square analyses were executed to determine the association between the farmer group and the biosecurity behaviour indicators. To determine if participation in the scheme affected farmers perception of the control behaviours effectiveness a mixed design Analysis of Variance (ANOVA) was performed with between factor variable Group (In-Scheme vs. Out of Scheme) and within factor variable Effectiveness of the biosecurity measures employed (Closed herd, Disinfecting equipment/Foot baths, Good husbandry, Herd Health Plan, Isolating new livestock, Purchasing livestock from a reputable source, and Vaccination). Participants in the In-Scheme and the Out of Scheme groups were asked to rate the effectiveness of each biosecurity measure using five point rating scale (1 = ‘not at all effective’, 2 = ‘not effective’, 3 = ‘neither effective, nor ineffective, 4 = ‘effective’, 5 = ‘very effective’). Significance was taken at the *p* = 0.05 level. The data met core assumptions of a normal data distribution and homogeneity of variance, for both between subject effects (perceptions of biosecurity measure effectiveness) and within subject effects (in-scheme vs. out of scheme).

A ‘STROBE’ Statement is included in the Supplementary Material [S1 Checklist].

### Limitations and cautions

Applied studies of human behaviour tend to suffer a number of well-known limitations. First, such work tends to rely on self-reporting, which means that participants may simply relay ‘the perceived correct answer’ as opposed to the actual behaviour undertaken. Second, sampling often relies on convenience or ‘snow balling’ approaches, which may lead researchers to individuals who may or may not be reflective of the population under study.

To decrease the potential influence of the farmers’ response bias Jones [25] or/and the ‘observer effect’ questions regarding bio-security behaviours and BVD were kept intentionally neutral (the questionnaire was evaluated by a linguist to remove any elements that may be perceived by enumerators or respondents as ‘leading’ or that which unintentionally may draw out/influence a response). Core attitudinal and behavioural questions were rephrased to enable participants to further elaborate upon specific issues. This redundancy was built in to enable the researchers to evaluate the consistency, accuracy and in some cases, the truthfulness of responses. For example, when farmers offered that ‘a closed herd’ was the best approach to disease control, a range of alternatively worded questions on this and a range of other bio-security behaviours were included across the interview to better determine if this was an actual behaviour or stated belief by the farmer involved.

Clearly on farm verification of actual, as opposed to reported behaviours, would alleviate some but not all of these issues with self-reporting. Indeed, such visits may be equally prone to the biases above i.e. some bio-security behaviours may or may not be visible on the day.

Convenience sampling may also be problematic in terms of generating conclusions applicable to a wider population. From the outset, this study makes no claims as to universality of attitudes and behaviours understudy. Rather, the reported attitudes and behaviours are explored in terms of their relation to a specific disease control scheme in a single geography. In this manner, the wider relevance of the study’s conclusions relate to disease control schemes and not the attitudes and behaviours under study per se.

## Results

Overall, the average age of both in-scheme farmers (*M* = 63.03, *SD* = 8.18) and out of scheme farmers was greater than 55 years, (*M* = 58.42, *SD* = 10.49) with in-scheme farmers forming the oldest subset of the study group, *t*(66) = 2.03, *p* = .047, *d* = 0.49. Moreover, the in scheme farmers (*M* = 169.28, *SD* = 135.84) reported to have larger herds than out of scheme farmers (*M* = 112.89, *SD* = 121.85), *t*(98) = 2.61, *p* = .010, *d* = 0.44.

Interestingly, participation in the scheme did not appear to impact the range of bio-security behaviours reported but did influence the adoption rates of particular actions. Not surprisingly, across both groups, the disease control measure mentioned with the highest frequency was a ‘closed herd’ (57% of the in scheme vs. 40% of the out of scheme farmers). A minority of farmers reported activities such as the ‘use of disinfectants’ and ‘isolating new stock’. Approximately 15% of farmers across both groups offered that ‘none’ of the existing disease control strategies were effective in controlling the spread of livestock disease (Table 1).

**Table 1:** Bio-security measures employed: In-scheme vs. out of scheme farmers.

Control measures	% mentioned In-Scheme (N = 53)	% mentioned Out of Scheme (N = 47)
Closed herd	57	40
Use of Disinfectants/Foot baths	8	19
Deworming	0	2
Good fencing	0	2
Herd Health Plan	6	2
Isolating new livestock	6	11
Milking hygiene	4	0
None	15	15
Vaccination	0	2
Good husbandry	2	2
Stock from reputable source	0	4

While it is not surprising that farmers trading at local markets (comprising most of the non-scheme members) were likely to prioritise ‘isolating new livestock’ and ‘the use of disinfectants’, most did not keep ‘closed’ herds despite the large number of mentions. To explore the finding further, farmers across both groups were asked to rank the disease control measures in terms of ‘effectiveness’ (where 1 = the most effective measure to 4, the least effective).

Keeping a ‘closed herd’ was ranked as first in effectiveness by both in-scheme (55%) and out of scheme (38%) farmers. While ‘vaccination’ was deemed best by more out of scheme farmers (13%) than, the six percent of in-scheme study participants who were required to vaccinate as part of the BVD scheme. Alternatively ‘use of disinfectants’ ranked second by the in-scheme (25%), and third by over 10% of the out of scheme farmers. Interestingly, implementing no control measures was ranked fourth by 81% of out of scheme farmers, while ‘good husbandry’ was linked to disease control across both groups (Tables 2 and 3).

**Table 2:** In-scheme farmers’ ranking of disease control measures (by effectiveness).

	In Schee group (N = 53) % ranking			
Control measures	1	2	3	4
Closed herd	62	11	9	0
Use of Disinfectants/Foot baths	9	25	4	4
Deworming	0	0	2	4
Good husbandry	6	19	8	4
Herd Health Plan	2	2	2	0
Isolating new stock	4	14	12	10
Milking hygiene	4	2	2	0
None	4	0	0	0
Purchasing livestock from a reputable source	0	6	2	2
Vaccination	6	4	8	0
No response	0	14	48	77

**Table 3:** Out of scheme farmers’ ranking of disease control measures (by effectiveness).

	Out of Scheme group (N = 47) % ranking			
Control measures	1	2	3	4
Closed herd	49	19	6	2
Common sense	0	2	0	0
Use of Disinfectants/Foot baths	13	4	13	2
Deworming	0	11	2	2
Good husbandry	6	4	12	11
Herd Health Plan	2	0	0	0
Isolating new stock	6	20	18	2
Milking hygiene	0	0	0	0
None	6	4	2	81
Purchasing livestock from a reputable source	4	0	0	0
Vaccination	13	4	0	0
No response	0	25	47	0

When farmers were asked the main strategy they *actually* employ to control BVD on their farms the majority of farmers reported having a ‘closed herd’ (66% in-scheme vs. 56% out of scheme farmers), and/or ‘disinfecting”, which includes foot baths and disinfecting vehicles upon entry to the farm (22% in-scheme vs. 13% out of scheme farmers). A further 2% of the out of scheme farmers reported that they were ‘testing for BVD’ while 17% reported ‘vaccinating’ and 6% offered that ‘buying stock from a reputable source’. Nevertheless, many of the farmers at the markets were buying animals, thereby the notion of a ‘closed herd’ (where new animals are not brought in) while believed to be the ‘safest’ with regard to disease exposure was not being practiced in its purest form. Hence, farmers who purchased from a ‘reputable source’ often still categorised their herds as ‘closed’.

Other variables that could potentially influence the choice of biosecurity behaviours farmers adopt are gender and age. As the study set was biased toward men (only two women farmers were interviewed) we explored the association between age groups: from 20 to 50 years vs. from 51 to 80 years and the main BVD control behaviours farmers employed (Table 4).

**Table 4:** BVD control behaviours adopted: Younger vs. older farmers.

Control measures	% mentioned Younger farmers (20 to 50 years) (N = 32)	% mentioned Older farmers (51to 80 years) (N = 68)
Closed Herd	45	47
Use of Disinfectants/Foot baths	10	18
Good husbandry	10	4
Herd Health Plan	3	4
Isolating new livestock	16	7
Purchasing livestock from a reputable source	7	0
Vaccination	6	12
None	3	7

Statistical analysis using a chi-square test found no overall association between the age and the control measures adopted *X*^*2*^(7) = 9.16, *p* = .24. A majority of both groups: younger (45%, *N* = 14) and older (47%, *N* = 32) reported adopting a ‘closed herd’ to control BVD.

**Fig 1:**
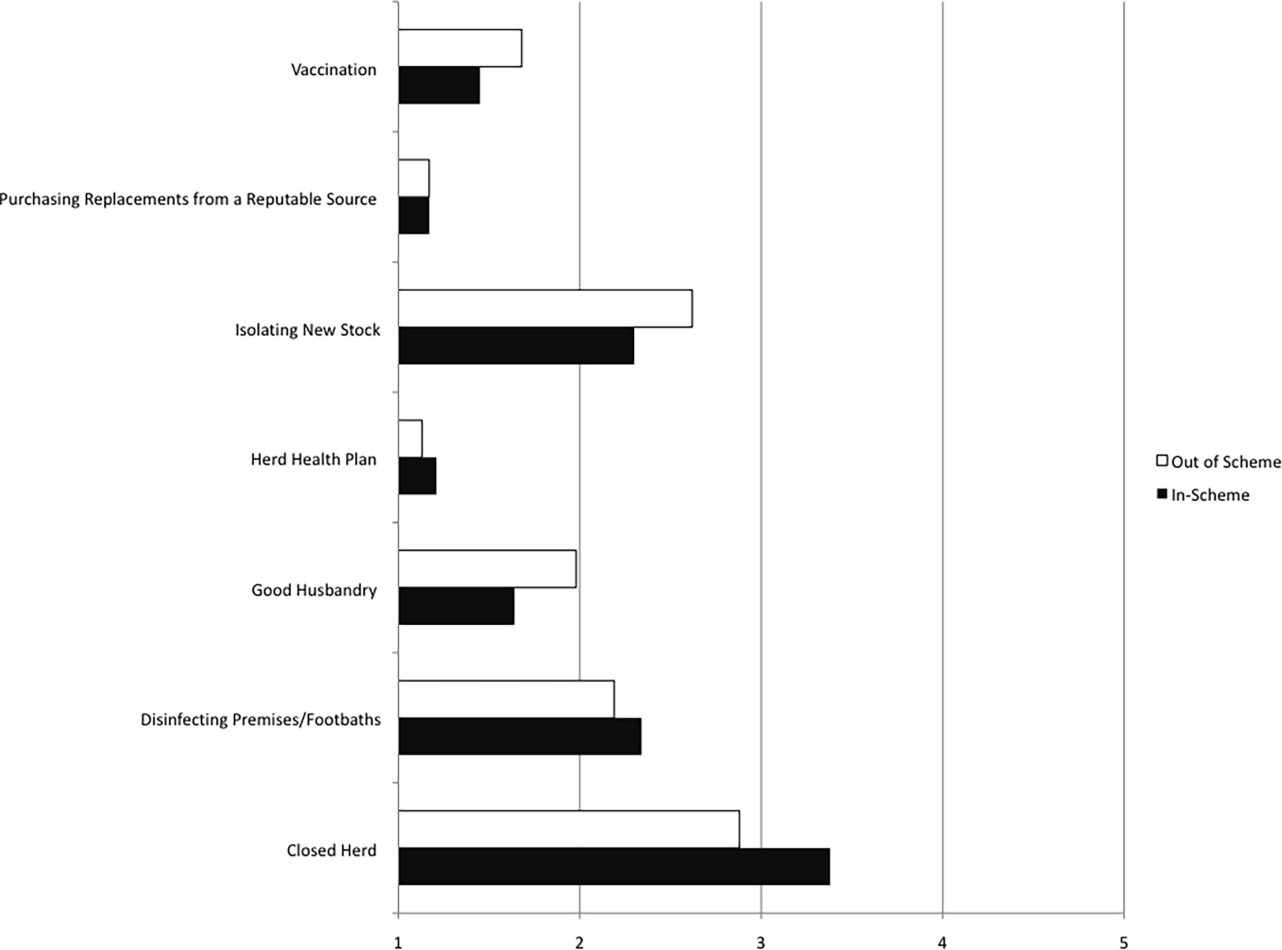
In-scheme vs. out of scheme farmers disease control behaviours effectiveness rating. Solid black bars indicate the in-scheme farmers’ effectiveness ratings of the disease control behaviours. Solid white bars show the out of scheme farmers’ ratings. Effectiveness ratings ranging from 1 = ‘not at all effective’ to 5 = ‘very effective’.

To determine if participation in the scheme affected farmers perception of the control behaviours effectiveness a mixed design Analysis of Variance (ANOVA) was performed with the ‘between factor’ variable (In-Scheme vs. Out of Scheme) and ‘within factor variable’ the effectiveness of the biosecurity measures employed: closed herd, using disinfectants, good husbandry, having a herd health plan, isolating new stock, purchasing livestock from a reputable source, and vaccination. The analysis revealed no significant interaction between Group membership and Effectiveness Ratings, *F*(6, 588) = 1.286, *p* = .31, η^2^ = 0.013. Thus, taking part in the scheme had little impact on farmers’ perception of the effectiveness of particular disease control behaviours (Fig 1).

### Disease priorities

All participants were asked to freely list those diseases they felt were the biggest priority or problem for their farming operation. Overall, the majority of in-scheme farmers (68%) reported ‘not having’ a priority disease (Table 5). Nevertheless, across both groups bovine tuberculosis was a concern with out of scheme farmers also worried about a reoccurrence of foot and mouth disease.

**Table 5:** Livestock disease priorities.

Disease priority	% mentioned In Scheme (N = 53)	% mentioned Out of Scheme (N = 47)
Black Leg	0	4
Bluetongue	6	4
Bovine Viral Diarrhoea	4	6
Foot and Mouth Disease	0	11
Johne’s Disease	0	4
Leptospirosis	0	2
Liver fluke	0	2
Mastitis	2	2
None	68	21
Pneumonia	6	4
Bovine Tuberculosis	14	38
Total	100	100

Statistical analysis showed no significant association between the taking part in a scheme and prioritising BVD *X*^*2*^(1) = 0.36, *p* = .55. Only 4% of the in-scheme and 6% of out of scheme farmers reported BVD to be a priority disease (Table 5).

The responses further revealed that a large proportion of the interviewed farmers (96% of the in-scheme farmers and 97% of the out of scheme farmers) shared the belief that BVD was no longer a threat.

ID 45: ‘BVD is under control’.

ID 42: ‘BVD is almost sorted out now’.

ID 51: ‘All steps have now been taking to address the [BVD] problem’.

ID 22: ‘BVD is the problem of the past’.

However, livestock disease in general and BVD in particular is often subject to social [13]. Indeed, despite being a member of a BVD control scheme, only three farmers across the study set mentioned that they shared BVD status information with their friends and neighbours. Reasons for not disclosing BVD status largely revolved around the notion that BVD was a ‘dirty’ disease and hence a reflection of a farmer’s husbandry skills:

ID 96: ‘…farmers reluctant to share, if haven't got it [BVD] they're not worried…They think will lose more than will gain—dirty farm syndrome. Pedigree breeders have valuable stock and don't want to disclose it.’

ID 88: ‘People worried who will use it [BVD status] in the future’.

ID 33: ‘[BVD viewed]…in a bad light. Speak to each other, but wouldn't trust what they said though.’

Wider animal health issues were also not disclosed, 75% of the in-scheme farmers and 72% of out of scheme farmers noted that they were too embarrassed or/and ashamed to discuss livestock disease.

ID 94: ‘No farmer likes to admit to have a problem [on their farm] because it is not only reflects badly on my farm image but it is also too embarrassing for a farmer.’

ID 13: ‘I am embarrassed to discuss my farm problems.’

ID 72: ‘I never talk to anyone, because I do not want people to know problems on my farms. Ego thing, you know.’

ID 87: ‘I do not want to admit to have a problem on my farm as it reflects badly on my reputation.’

Membership in a group broadly entails that members share a common aim and will work together to meet that aim. Clearly in relation to a disease eradication scheme, it would be expected that group members would desire to be free of the disease on at least the farm level.

### Motivation for scheme membership

**Fig 2:**
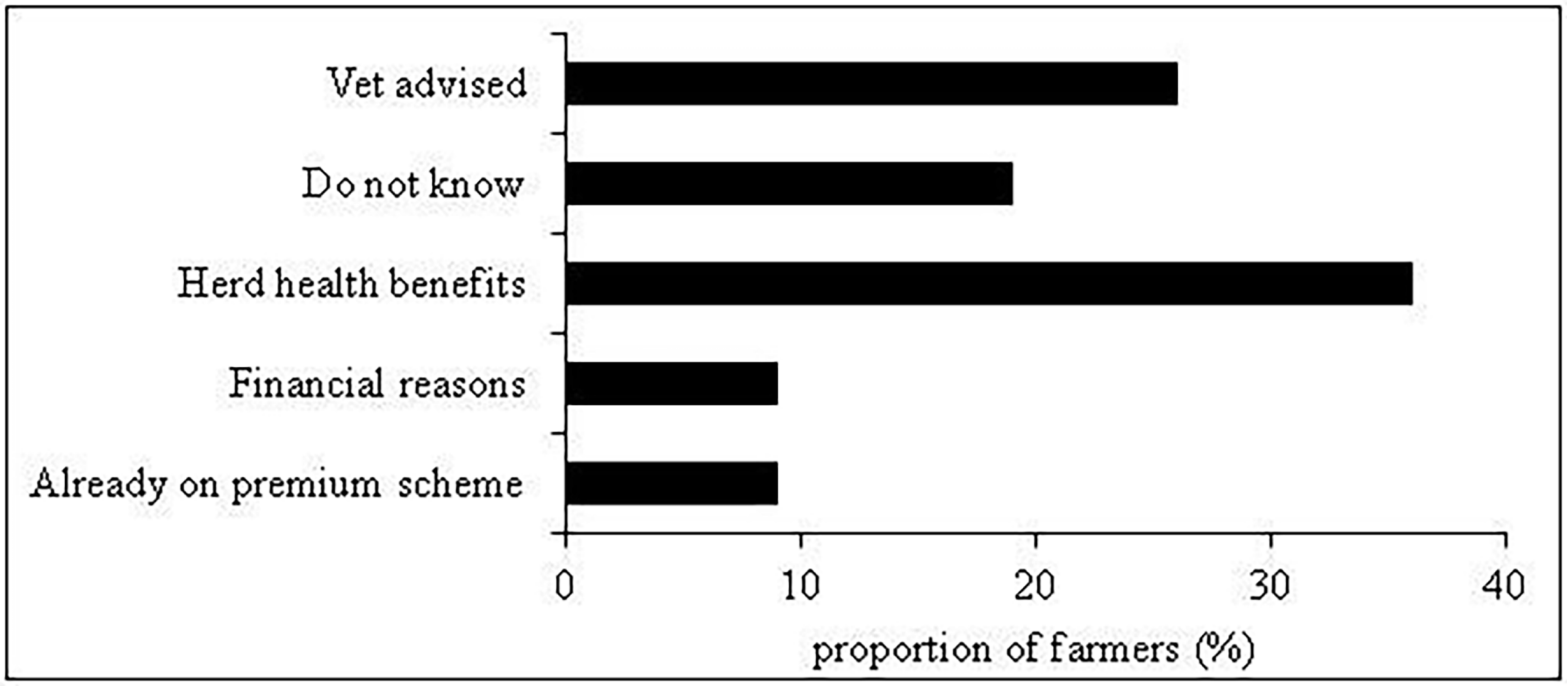
In-scheme farmers: Reasons offered for joining the BVD scheme (percent response).

When asked why they decided to join the scheme, 20% of farmers did not offer a particular reason. Nearly 1/3^rd^ (29%) simply stated that they joined the scheme because ‘their vet advised them’. Despite not admitting to a problem with BVD in the first place, 35% of farmers stated that they took part in the scheme because they either ‘wanted to solve an existing problem’ (25%) or for ‘financial reasons’ (10%). A further 25% reported to get involved in the scheme because they wanted to ‘improve health of their animals’ (Fig 2).

**Fig 3:**
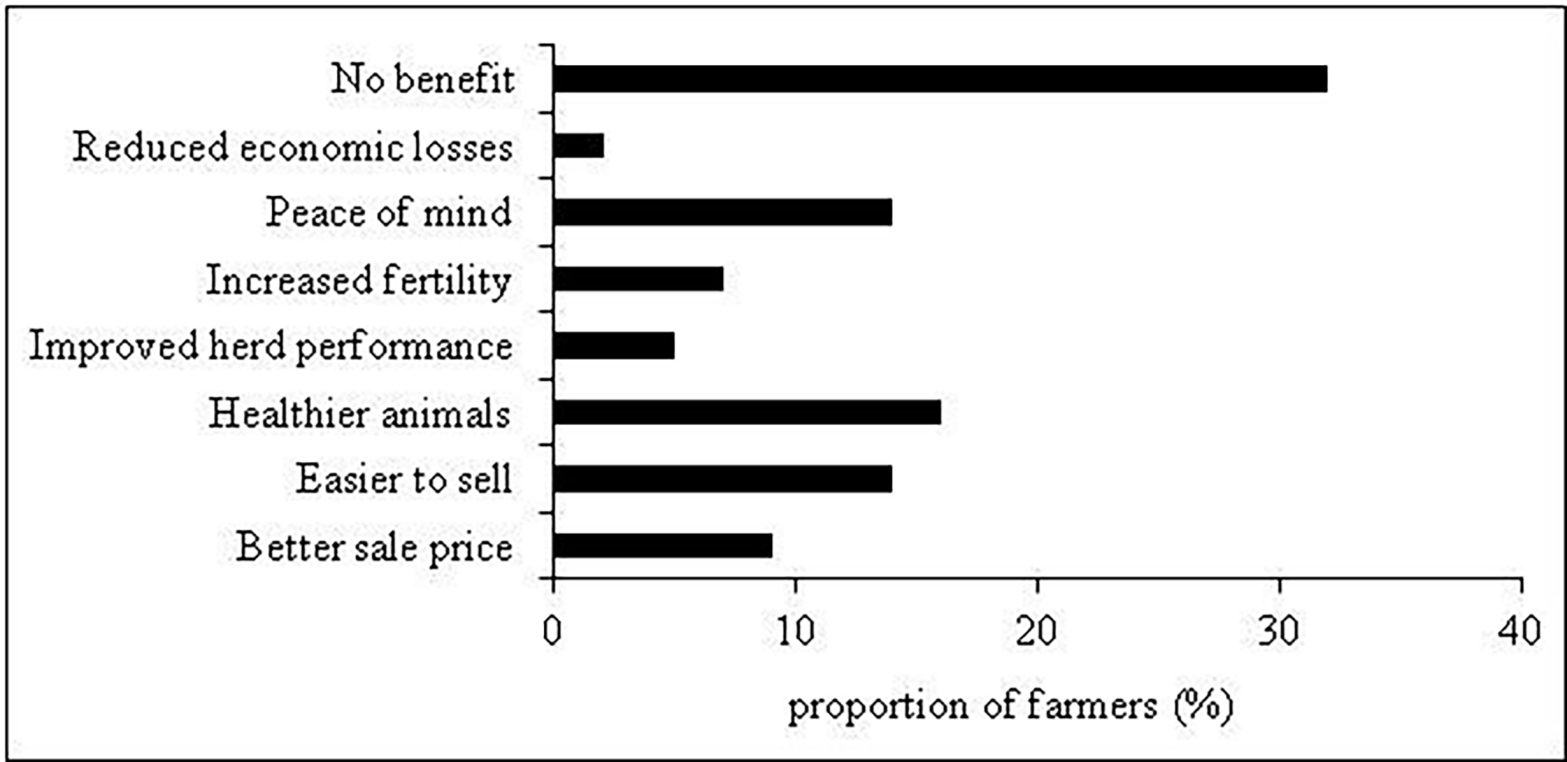
Frequency of training course attendance by the in-scheme and out of scheme farmers.

In contrast, only 6% of out of scheme farmers believed that a scheme could offer some benefits (Fig 3).

Despite declaring that BVD was ‘no longer a problem’ or a ‘priority’, 22% of farmers noted that scheme membership offered psychological benefits i.e. ‘peace of mind’ (Table 6). A variety of economic benefits were offered ranging from ‘increased fertility’ to ‘easier to sell/better sale price’ and ‘better herd performance’ (Fig 3).

**Table 6:** Behaviours adopted by in-scheme farmers who reported the scheme offered psychological and/or economic benefits.

Disease control behaviours adopted	% mentioned Economic benefits (N = 27)	% mentioned Psychological benefits (N = 9)
None	0	11
Closed herd	59	67
Use of Disinfectants/Foot bath	11	0
Good husbandry	4	11
Herd Health Plan	4	11
Isolating new stock	11	0
Vaccination	11	0

When association between the type of scheme benefits reported by the in-scheme farmers (i.e., psychological benefits and economic benefits) and the type of disease control behaviours adopted was examined no significant association was found between these variables *X*^2^(6) = 7.39, *p* = .29.

Moreover, the chi-square analysis found no association between the type of the scheme benefits reported by the in-scheme farmers (i.e. psychological and economic benefits) and disease priority (i.e. BVD or other diseases) *X*^2^(2) = 1.01, *p* = .58. Only 7% of in-scheme farmers who stated the BVD scheme offered economic benefits (N = 27) and none of the in-scheme farmers who reported to benefit from the scheme psychologically (N = 9) reported BVD to be a priority disease.

Clearly the reasons for joining a scheme are likely to influence a farmer’s decision to continue membership over time. Thus, it is reasonable to assume if these expectations are not fulfilled the membership is likely to discontinue.

### Knowledge acquisition

Statistical analysis showed a significant association between taking part in the scheme and whether or not farmers had attended a training course *X*^2^(1) = 5.50, *p* = .027. While the majority of in-scheme farmers (57%) reported attending a training course, only 33% of the out of scheme farmers noted they had gone on a course.

Across scheme members, a larger proportion of interviewed farmers (28%) attended courses ‘annually’. Conversely non-members, reported much lower attendance rates with ‘occasionally’ (9%) or only ‘once’ (11%).

Interestingly, 36% of farmers who reported attending a training course(s) (18% in-scheme and 18% of the out of scheme group farmers) were not able to recollect what was actually discussed on the courses they attended. Almost half (48%) scheme members said that the ‘general animal health’ (18%), ‘lameness’ (18%) and ‘livestock fertility’ (12%) were discussed on those courses they took part in. Conversely, the topics of the courses attended by non-scheme members included ‘animal health’ (36%), ‘fertility’ (9%), ‘foot trimming’ (9%) and ‘liver fluke’ (9%). Only 6% of the in-scheme farmers and none of the out of scheme farmers who went to a training course(s) recalled that BVD was mentioned on the training courses they attended (Table 5).

## Discussion

Farmers participating in the study were, at least ostensibly, from two very different mindsets: those electing to be part of a scheme sponsored by their veterinary practice to those willing to speak to researchers on a busy market day and out with any such formal disease control programme. As such, from the outset, the expectation would be that these groups would have very different attitudes toward disease control in general and BVD control more specifically. Interestingly, in this case, it was the similarities in stated attitudes and behaviours that proved to be more relevant than the differences.

For example, we did not find an association between participation in the BVD scheme and farmers’ priority livestock disease, motivation, or knowledge acquisition in relation to BVD. Scheme membership equally did appear to influence perceptions regarding the importance of specific bio-security measures employed. Membership was, however, associated with the training course attendance and the priority accorded to wider biosecurity behaviours such as having a ‘closed herd’ and ‘the use of disinfectants’.

Indeed, when the in-scheme and the out of scheme farmers were asked to identify the measure(s) that in their opinion would be the most effective in controlling BVD, a ‘closed herd’ was viewed by farmers from both groups as the best measure to control BVD. Every sixth farmer across the study set reported ‘none’ of the existing BVD control measures to be effective. In-scheme farmers, did not view vaccination as a critical BVD control measure, despite its inclusion in many of the scheme-led ‘bespoke biosecurity plans’ developed by the vets for the farmers involved.

In contrast to Toma et al. [14] we found scheme membership did not increase the number of BVD control measures farmers reported to employ. Rather, across the two groups, more out of scheme farmers reported using a greater number of BVD control measures (i.e., ‘closed herd’, ‘disinfecting’, ‘BVD testing’, ‘vaccination’ and ‘purchasing only from known sources’) than in-scheme farmers (i.e., ‘closed herd’ and ‘disinfecting’). While the authors did not verify if such answers were the ‘perceived correct ones’ or reflected actual on-farm practice, the finding does illustrate the ongoing need for knowledge transfer across disease control schemes. Further, farmers’ ratings of effectiveness also illustrated that taking part in the scheme did not appear to influence farmers’ perception of the effectiveness of particular biosecurity behaviours. In both groups farmers reported ‘closed herd’ and ‘disinfecting’ to be ‘effective’ or/and ‘very effective’ BVD control behaviours. Further the biosecurity behaviours farmers engaged in to control the BVD were not associated with farmer age: the majority of the older and the younger farmers offered that a ‘closed herd’ controlled the disease.

Equally surprisingly, participation in the scheme did not appear to influence the way farmers prioritized animal health constraints. Across the study groups only a small proportion of farmers reported BVD to be a priority. It is interesting to note that while BVD remains a clear threat in Norfolk and Suffolk counties, the majority of farmers in both groups reported BVD to be ‘no longer a threat’ or to be ‘under control’.

Both in-scheme and the out of scheme farmers found sharing information about animal health to be a very sensitive subject (with 36% of in scheme farmers and 34% of out of scheme farmers reported to share the information about animal health with members of their social network). The study also illustrated that perceptions regarding sensitive subjects such as animal health information sharing had a large emotional component. According to Heffernan et al. [13], BVD is largely perceived to be the fault of individual farmer and often entails social censure. Indeed, participants in both groups explained their choice to not share animal health information in order to avoid feeling embarrassed or ashamed.

The in-scheme farmers’ responses also demonstrated that the majority of farmers did not offer an explanation as to why they joined the BVD scheme or simply noted they joined the scheme because their local vet advised them to. The finding illustrates the importance of vets in the frontline of bio-security engagement. Yet when the in-scheme farmers were asked to list scheme benefits, a high percentage of farmers deemed the scheme to have no benefits at all. Such responses reveal an important lesson for future disease control schemes: there is an urgent need for veterinarian-led, on-going knowledge transfer that supports continued farmer engagement across the project cycle. The findings demonstrate that while the role of veterinarians in promoting disease control activities is undisputed, the manner and means by which farmers are engaged could be improved.

In relation to knowledge, scheme membership did increase the frequency with which training courses were attended. While such course attendance did not appear to influence the adoption of specific biosecurity behaviours, the finding does illustrate that in-scheme farmers were more open to self-learning. And in this manner, augmenting disease control programs with specific training activities both within and external to the scheme itself may enhance overall effectiveness.

## Conclusion

Organising farmers into groups to control livestock disease and ultimately, to support behavioural change has historical precedence [26, 27]. Part of the problem is there are few alternatives: volunteer groups are seen as cost-effective and forming such groups is viewed as positive direct action against disease threats. Yet how successful such groups are in meeting either of the above objectives has not been the subject of much research interest.

Our findings also demonstrate the complexity in assessing the impact of disease control schemes on farmer behaviour. As noted above, across the groups, many of the attitudes and behaviours toward BVD and disease control more widely, were the same. While scheme farmers were more open to knowledge transfer activities, changing the lens by which they viewed BVD did not appear to be completely successful.

In this manner, the study offers three policy relevant cautions. First, the findings suggest that efforts to better identify and understand the differences between farmers groups may be counterproductive in terms of disease control. The similarities between in-scheme and out of scheme farmers in terms of attitudes and behaviours far outweighed the differences. Second, to enhance the impact of disease control schemes, components that address core farmer beliefs and behaviours, must be better integrated across programme activities. Finally, the social factors regarding BVD i.e. notions of the disease as ‘dirty’ and the censure received with a positive diagnosis must not be overlooked. In this manner, it is clear that a more holistic approach to disease control that accounts for and addresses critical attitudinal and behavioural barriers at the farm level are likely to improve the impact of disease control schemes.

But the findings also leave us with a related question for future research: is it time to look beyond farmers groups for disease control?

## Supporting information

S1 Checklist Strobe checklist. (DOC)
